# Paddle leads for the treatment of nonsurgical back pain—The DISTINCT study

**DOI:** 10.1111/papr.70033

**Published:** 2025-04-23

**Authors:** Steven Falowski, Michael J. Dorsi, Robert Heros, Rafe Sales, Edward Tavel, Todd Lansford, Martin E. Weinand, Nrupen Baxi, Jason Garber, James Forage, Albert E. Telfeian, Haddad Souheil, Christopher Gilligan, Timothy Deer, Robert Levy, Jessica Jameson, Susan Moeschler, Jonathan Duncan, Chi Lim, Mehul Desai, Julie Pilitsis, Udoka Okaro, James Yue

**Affiliations:** ^1^ Neurosurgical Associates of Lancaster Lancaster Pennsylvania USA; ^2^ Neurosurgery, University of California Los Angeles Los Angeles California USA; ^3^ Spinal Diagnostics Tualatin Oregon USA; ^4^ Summit Spine Institute Portland Oregon USA; ^5^ Clinical Trials of South Carolina Charleston South Carolina USA; ^6^ South Carolina Sports Medicine and Orthopaedic Center Charleston South Carolina USA; ^7^ Banner University Medical Center Tucson Arizona USA; ^8^ Department of Neurosurgery Montefiore Medical Center and Albert Einstein College of Medicine Bronx New York USA; ^9^ LVNI Center for spine and brain Surgery Las Vegas Nevada USA; ^10^ The Spine and Brain Institute Nevada USA; ^11^ Department of Neurosurgery, Warren Alpert Medical School Brown University Providence Rhode Island USA; ^12^ Saint Louis Pain Consultants Creve Coeur Missouri USA; ^13^ Brigham & Women's Hospital Boston Massachusetts USA; ^14^ The Spine and Nerve Center of the Virginias Charleston West Virginia USA; ^15^ Anesthesia Pain Care Consultants Tamarac Florida USA; ^16^ Axis Spine Center Post Falls Idaho USA; ^17^ Department of Anesthesiology and Perioperative Medicine, Mayo Clinic Rochester Minnesota USA; ^18^ Burkhart Research Institute for Orthopaedics San Antonio TX USA; ^19^ Carolina Orthopaedic & Neurosurgical Associates Spartanburg South Carolina USA; ^20^ International Spine, Pain & Performance Center Washington DC USA; ^21^ Department of Neurosurgery University of Arizona Tucson Arizona USA; ^22^ Abbott Labs Austin Texas USA; ^23^ Frank H. Netter School of Medicine Quinnipiac University, Connecticut Orthopaedics Hamden Connecticut USA

**Keywords:** low back pain, spinal cord stimulation

## Abstract

**Introduction:**

DISTINCT is a randomized controlled trial evaluating passive recharge burst SCS compared with CMM in improving pain and pain‐related physical function in patients suffering from chronic back pain without prior lumbar surgery, and for whom corrective surgery is not an option. Sub‐analyses of outcome measures are presented for a subset implanted with paddle leads at both 6 and 12 months.

**Objective:**

To investigate the treatment effect of using BurstDR‐capable SCS in nonsurgical low back pain (NSLBP) patients with paddle leads.

**Methods:**

An independent board‐certified spine surgeon reviewed each case, confirming a lack of corrective surgical options. Out of 29 sites and 115 implants, 10 sites implanted 50 patients with paddle leads. Primary and secondary endpoints assessed improvements in low back pain intensity (NRS), low back pain‐related disability (ODI), pain catastrophizing (PCS), and patient global impression of change (PGIC).

**Results:**

Data for patients with NSLBP and implanted with paddle leads are available for 47 and 44 patients at 6 and 12 months, respectively. Patients reported significant reductions in pain relief at 6 and 12 months, decreasing from 7.8 ± 1.2 at baseline to 2.0 ± 1.6 and 2.2 ± 2.2, respectively (*p* < 0.0001). Disability was substantially reduced (> 20 points) from severe to mild at 6 and 12 months; ODI reduced from 54.4 ± 15.0 at baseline to 19.9 ± 14.1 and 22.1 ± 13.6, respectively (*p* < 0.0001). Pain catastrophizing improved to reflect the average of a non‐chronic pain population; decreased from 27.6 ± 13.1 at baseline to 8.1 ± 8.2 and 7.8 ± 9.9 (*p* < 0.0001). 93.2% of patients reported a moderately better‐much‐improved outcome on PGIC. Ten complications occurred in 9 patients, 3 being severe device‐related events. Two explants were required; one due to infection and one due to persistent pain at the IPG site, and one lead‐related event was resolved by surgical repositioning.

**Conclusions:**

Passive recharge burst using paddle leads provides substantial relief to patients suffering from severe, debilitating, NSLBP. Patients report significant improvements in pain, function, and pain‐related emotional distress. In addition, the rate of adverse events is low, supporting a favorable safety profile.

## INTRODUCTION

Spinal cord stimulation (SCS) is indicated to treat chronic, intractable pain of the trunk and/or limbs, including unilateral or bilateral pain associated with failed back surgery syndrome, nonsurgical back pain (without prior surgery and not a candidate for back surgery), and diabetic peripheral neuropathy of the lower extremities. Patients may be implanted with a percutaneous or paddle lead.^1^ Paddle leads are reported to offer some advantages over percutaneous cylindrical leads. The larger size and design of paddle leads are reported to offer broader coverage of the dorsal columns, resulting in improved paresthesia coverage and improved pain relief when compared to percutaneous leads. Other purported advantages include increased targeting of specific areas of the spinal cord, improved quality of life, and reduced healthcare utilization.^2^, ^3^, ^4^, ^5^ Recent publications show that paddle lead stimulation can be customized to patient pain patterns using multisite BurstDR programming, improving patient outcomes.^6^, ^7^, ^8^ Paddle leads are also considered safe, with low complication rates such as lead migration and fracture.^9^, ^10^

Patients with painful conditions amenable to SCS are evaluated preoperatively to assess their suitability for SCS. This evaluation may include a thoracic spine MRI or CT to assess the patency of the spinal canal. Patients undergo an SCS trial lasting 4–7 days, generally using percutaneous electrodes and an external generator to evaluate therapy impact on chronic pain before a permanent implant.^11^ The trial leads are often implanted by a pain specialist. Patients who report at least 50% pain relief are generally considered suitable candidates for permanent implants.^12^ Permanent implants are done using a paddle or percutaneous leads by pain medicine specialists with percutaneous leads, while surgeons often prefer paddle leads. Paddle leads are placed via open laminotomy or laminectomy in the dorsal epidural space at the spinal level of maximal therapeutic benefit as determined during the trial.

Several studies report significant pain relief in patients implanted with paddle leads for various chronic pain conditions. Paddle leads demonstrate efficacy in providing long‐term pain relief, with some studies reporting sustained benefits over several years of follow‐up. SCS with CMM is cost‐effective compared with CMM alone in the management of Failed Back Surgery Syndrome (FBSS).^13^, ^14^, ^15^

The DISTINCT study is a multicentered, prospective randomized controlled trial that evaluated the efficacy of spinal cord stimulation (SCS) compared with conventional medical management (CMM) in improving pain and back pain‐related physical function in patients with chronic, refractory axial low back pain who had not undergone lumbar surgery and for whom surgery was not an option.^16^ This subgroup analysis focuses on assessing the patient selection, safety, and effectiveness of the DISTINCT Paddle cohort.

## STUDY OBJECTIVE

This analysis reports on the safety and performance outcomes for the DISTINCT Paddle leads patients.

### Methods

All study documents received institutional review board (IRB) approval prior to patient enrollment. The DISTINCT study is registered on ClinicalTrials.gov (NCT04479787). Consent was obtained from all potential patients prior to enrollment. The study is conducted in accordance with the US Code of Federal Regulations and the World Medical Association Declaration of Helsinki. The study enrollment, design, and outcomes are discussed in Deer et al. (2023).^16^ Magnetic resonance imaging (MRI) and/or computed tomography (CT) images of the spine obtained within 12 months were reviewed by an independent orthopedic spine surgeon prior to study enrollment to confirm the lack of an identifiable pathology that could effectively be treated with surgery. Study inclusion, exclusion, and enrollment details have been previously published.^16^ As part of the inclusion/exclusion criteria, all patients' primary pain was back pain, and patients reporting leg pain greater than back pain were excluded from the study.

Fifty patients are included in this analysis. All 50 patients were consented and enrolled before a 5–7 days SCS trial using a percutaneous lead and external pulse generator. Patients reporting ≥50% or more pain relief received a permanent implant with paddle leads and compatible IPG (Abbott Labs, Penta leads: 3228, Proclaim IPG: 3660). A single paddle lead was implanted with the paddle tip at spinal level T7, T8, or T9. All 50 patients were implanted under general anesthesia, with 33% using neuromonitoring. Trial leads for all 50 patients were implanted by pain specialists, while neuro/ortho surgeons implanted the permanent leads. The intraoperative lead location was driven by the location of the mapping during the trial procedure. Permanent procedures were performed either in a hospital or an ambulatory surgical center. Patients were monitored in the clinic at 1, 3, 6, and 12 months. For patients unable to access in‐clinic care, a telehealth appointment with remote programming of SCS devices occurred as needed (NeuroSphere Virtual Clinic, Abbott Labs, Plano TX).

Study outcomes included back pain relief (Numerical Rating Scale; NRS), disability (Oswestry Disability Index; ODI), catastrophizing thought (Pain catastrophizing scale; PCS), and patients' satisfaction (Patient Global Impression of Change; PGIC). Responders were defined as patients who observed a 50% pain improvement on the NRS scale and a 13‐point reduction on the ODI scale. Minimal clinically important difference (MCID) was defined as ≥2 on the NRS scale.^17^

### Results

**FIGURE 1:**
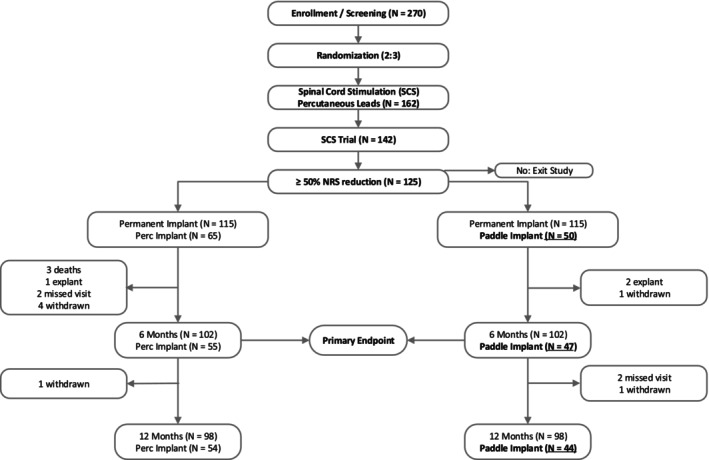
Patient consort diagram through 12 months of the DISTINCT study. The paddle population presented within this manuscript is bolded and underlined.

Patient Demographic: This analysis reports on 50 patients who were implanted with paddle leads—Figure 1 from the full 270 cohort. Female patients (31/50) made up a majority of the analyzed cohort—Table 1. The mean age ± SD of patients was 57.5 ± 11.9 years with a range of 32–87 years. At least 46% of patients were diagnosed with 1–2 indication(s) with 54% (*n* = 27) reflecting more than 2 diagnoses. Chronic, non‐specific, low back pain (72%) in addition to lumbar spondylosis (62%), lumbar radiculopathy (38%), lumbar spinal stenosis (22%), and lumbar facet arthropathy (20%) were the most common additional diagnoses. About 36% of patients reported having back pain and bilateral leg pain; 32.0% reported back pain and unilateral leg pain; and 32.0% of patients reported having back pain only (Table S1). Patients reported having pain for a mean of 13.79 ± 11.11 years, with the majority of the patients (68%, 34/50) reporting back pain in addition to unilateral (16/50) or bilateral (18/50) leg pain. All patients reported having previous physical therapy, while 51% (23/45) have used chiropractic/massage therapy. 95.8% of these patients have had injections in addition to 35.4% who also reported radiofrequency ablation/rhizotomy. About 42% of the patients reported ongoing opioid usage for an average MME Mean ± SD of 19.18 ± 14.19. During the duration of the study, 6 paddle patients were either explanted (2) withdrawn (2), or lost to follow‐up (2). Forty‐four patients completed the 12‐month follow‐up.

**TABLE 1:** Patient Demographics at Baseline.

	SCS (*N* = 50)
Age (year)	
Mean ± SD (*n*)	57.5 ± 11.9 (50)
Median (Q1, Q3)	59.0 (49.0, 67.0)
Gender, *n* (%)	
Female	62.0% (31/50)
Male	38.0% (19/50)
Race, *n*(%)	
White	78.0% (39/50)
Black or African American	10.0% (5/50)
Asian	0.0% (0/50)
American Indian or Alaska Native	2.0% (1/50)
Declined/Unable to disclose	8.0% (4/50)
Native Hawaiian or Other Pacific Islander	2.0% (1/50)
Pain Numeric Rating Scale (NRS)	
Mean ± SD (*n*)	7.9 ± 1.2 (50)
Median (Q1, Q3)	8.0 (7.0, 9.0)
Duration of patient's pain on patient's life (year)	
Mean ± SD (*n*)	13.79 ± 11.11 (50)
Median (Q1, Q3)	10.96 (5.00, 20.00)
Pain diagnosis*	
Chronic, non‐specific, low back pain	72.0% (36/50)
Discogenic pain	8.0% (4/50)
Degenerative disc disease	18.0% (9/50)
Lumbar disc herniation	4.0% (2/50)
Lumbar facet arthropathy	20.0% (10/50)
Lumbar radiculopathy	38.0% (19/50)
Lumbar spinal stenosis	22.0% (11/50)
Lumbar spondylosis	62.0% (31/50)
Mechanical low back pain	8.0% (4/50)
Spondylolisthesis	2.0% (1/50)
Scoliosis	4.0% (2/50)
Other	8.0% (4/50)
Paddle lead level implanted	
T7	32% (16/50)
T8	58% (29/50)
T9	10% (5/50)
Medication	
Opioids	42% (21/50)
MME mean ± SD	19.18 ± 14.19

*Physician/Patient may indicate more than one diagnosis.

**FIGURE 2:**
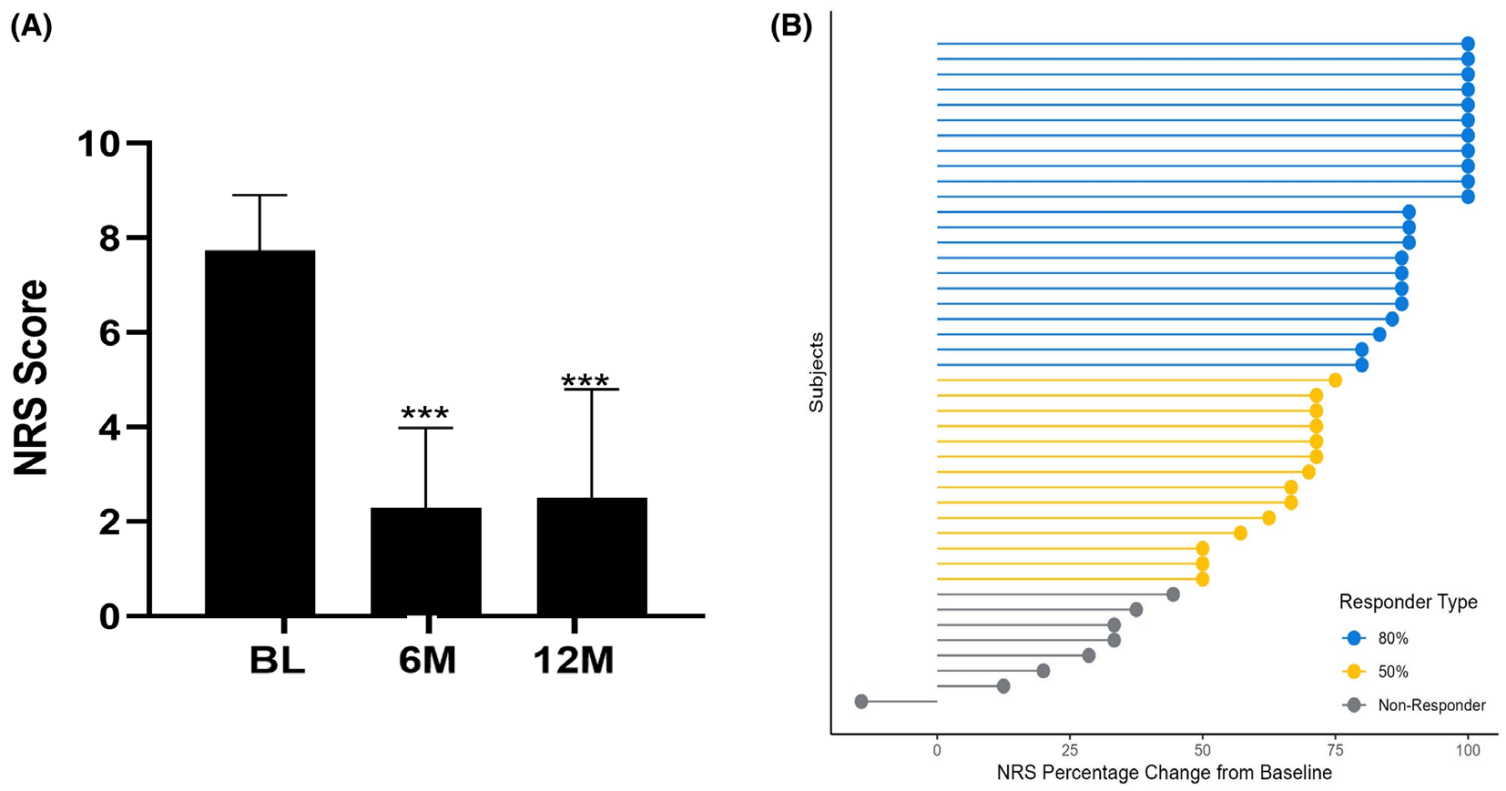
(A) Pain decreased from 7.8 ± 1.2 at baseline to 2.0 ± 1.6 at 6 M and 2.2 ± 2.2 at 12 M (*p* < 0.0001). (B) At 12 months, 82% of patients reported at least a 50% pain reduction. Additionally, 50% of patients reported an 80% pain reduction. (***indicates *p* < 0.0001).

Outcome Improvement: Eighty‐two percent (36/44) of patients reported at least a 50% pain improvement on the NRS scale (Figure 2). The pain decreased from 7.8 ± 1.2 to 2.0 ± 1.6 at 6 M, which remained clinically relevant at 12 M (2.2 ± 2.2, *p* < 0.0001) representing a 2.8x improvement above MCID (MCID is a 2.0 decrease or 30% improvement in pain).^18^, ^19^ Additionally, 50% of patients reported an 80% pain reduction. Except for 1 patient, 98% (43/44) of patients reported a pain improvement.

**FIGURE 3:**
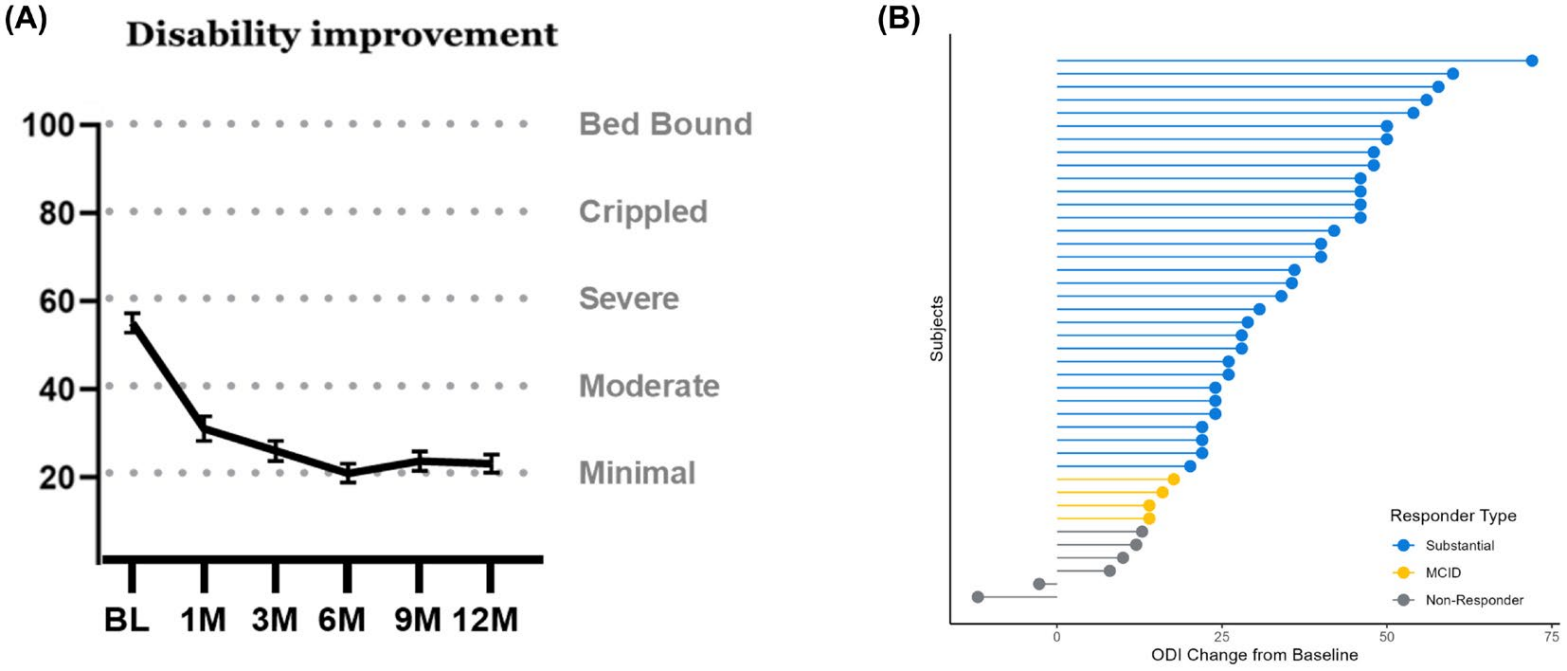
(A) 76% of the DISTINCT paddle population was classified as severely disabled at baseline. At 12 months, patients reported minimal disability. (B) 85.7% reported a ≥ 13 pt. improvement for MCID after 12 months of permanent implant. About 76.2% of patients reported a substantial improvement of 20 pt. decrease.

Likewise, 85.7% of patients reported a ≥ 13 pt. meaningful improvement in disability at 12 months (Figure 3). Patients previously reported severe disability (54.4 ± 15.0) at baseline, which decreased to minimal disability 12 months post‐permanent implant (22.1 ± 13.6) with 76.2% reporting a substantial improvement of 20 pt. decrease. All disability improvement was sustained through 12 months.

All patients in the study had predominantly axial low back pain, with subgroups showing variable amounts of unilateral or bilateral leg pain. A subgroup analysis of these distinct phenotypes reveals some differences, although the study was not powered for this. At 12 months, patients with back and unilateral leg pain, making up 32% of the sample population, reported 81% pain improvement and 62% functional improvement. Patients with back and bilateral leg pain, 36% of the sample population, reported 78% pain improvement and 64% functional improvement. Patients with only axial back pain made up 32% of the sample population and reported pain improvement and functional improvement of 54% and 50%, respectively. While all subgroups demonstrated pain relief from 54% to 81% at 12 months, the group with leg symptoms reported the best results (Table S1). It is also worth noting that patients whose primary complaint was leg pain or whose leg pain was greater than their back pain were excluded from this study.

**FIGURE 4:**
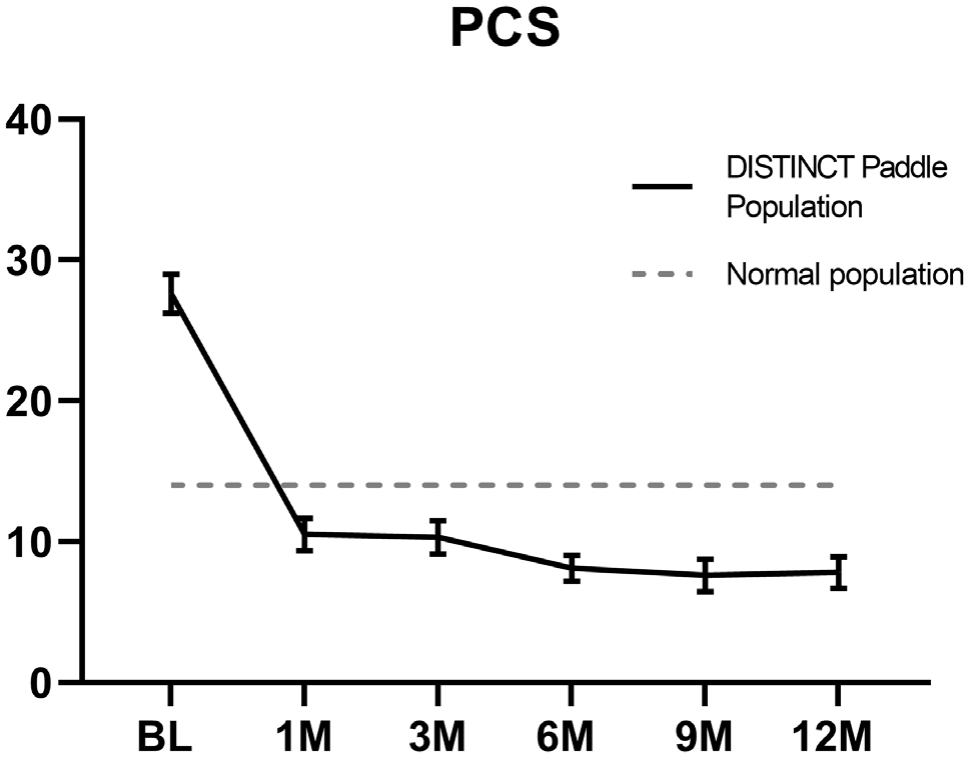
Pain catastrophizing improved to reflect the average of a non‐chronic pain population^20^; decreased from 27.6 ± 13.1 at baseline to 8.1 ± 8.2 and 7.8 ± 9.9 (*p* < 0.0001) reflecting that all patients indicate the population norm.

Patients reported an improvement in PCS from a baseline score of 27.6 ± 13.1 to a score at 12 months of 7.8 ± 9.9 (*p* < 0.0001) which reflects the score of a non‐chronic pain population^20^ – Figure 4. Patients reported an improvement in all outcomes—Table 2. A comparison of the DISTINCT study population for improvement outcomes between patients who received paddle or percutaneous leads shows no significant difference in outcomes between the two groups at 12 months—see supplemental data (Table S2).

**TABLE 2:** Patient‐reported outcomes.

Outcome	BL			3 M			6 M			12 M			*p* value
	Average	STD	*N*	Average	STD	*N*	Average	STD	*N*	Average	STD	*N*	
NRS	7.9	1.2	50	2.6	2.2	49	2	1.6	47	2.2	2.2	44	<0.001
ODI	54.4	15	50	22.4	14.6	47	19.9	14.1	45	22.1	13.6	42	<0.001
PCS	27.6	13.1	50	9.3	9.9	49	8.1	8.2	47	7.8	9.9	44	<0.001

Overall, 93.2% of patients reported a moderately better–much‐improved outcome on the Patient Global Impression of Change (PGIC) with a 93.7% satisfaction rate with their treatment.

Safety: Ten adverse events occurred in 9 patients‐Table 3. One event, a non‐severe event (dermatitis and desquamation) occurred during the trial implant. Permanent implant device‐related events accounted for 7 events‐3 severe adverse device events (2 infections and 1 post‐surgical pain) and 4 non‐severe device events (Implant migration, dermatitis and desquamation, infection, and pain at the IPG Site). A total of 3 device‐related events were surgically resolved (2 explants (4%) and 1 surgical revision (2%)). One hospital outpatient subject had an infection leading to a system explant, and persistent pain at the IPG site led to one system explant at an ambulatory surgical center (ASC).

**TABLE 3:** Reported adverse events and resolution.

Event description	AE classification	Location of procedure	Resolution
Severe adverse device event (SADE)			
Infection	SADE	Hospital outpatient	System explant
Post‐surgical pain	SADE	Hospital outpatient	Medication
Infection	SADE	Hospital outpatient	Medication
Adverse device effect (ADE)			
Implant migration	ADE	Ambulatory Surgery Center	Surgical revision
Dermatitis and desquamation	ADE	Hospital outpatient	Medication
Infection	ADE	Ambulatory Surgery Center	Medication
Persistent pain at the IPG site	ADE	Ambulatory Surgery Center	System explant
Severe adverse event (SAE—Not device‐related)			
Bone fracture	SAE	Hospital outpatient	Surgical
Pneumonia	SAE	Hospital outpatient	Medication
Congestive heart failure	SAE	Ambulatory Surgery Center	Medication

Three severe adverse events (1 bone fracture, 1 pneumonia, 1 congestive heart failure) adjudicated as non‐device/procedure‐related events were reported during this study. One implant migration leading to a system revision was reported for an ASC implant. The overall safety ratio, while not significantly different, was 2:3 (ASC:Outpatient).

Overall, this cohort reported an acceptable paddle lead adverse event rate, with all adverse events successfully resolved.

### Discussion

The DISTINCT study represents a unique opportunity to prospectively study a population of low back pain patients in expert centers working under two closely related paradigms; (1) trial done by pain medicine specialists (with percutaneous electrodes)—with the implant done by spine surgeons (ortho‐spine and neurosurgical) using paddle electrodes, and (2) pain medicine specialists who perform trials and their implant procedures using percutaneous electrodes—see Figure 1. The pain specialist‐spine surgeon paradigm has been called the “hub and spoke” model of interdisciplinary care by some, and the details of this paddle population are presented. The overall DISTINCT manuscripts present the aggregated data of both populations.^16^

Back pain in general is a burden both for patients and the healthcare systems, and is a leading cause of disability worldwide.^15^ The DISTINCT paddle population consists of patients with refractory pain who have failed to achieve adequate pain relief from conservative treatments including medications, physical therapy, and injections.^16^, ^21^ About 14 (28%) of the DISTINCT patients previously reported that pain interfered with their ability to work with 18% identifying as disabled. Six months after permanent SCS implant, the majority of patients reported minimal disability demonstrating the efficacy of paddle leads in reducing disability and the economic impact of indirect costs such as lost wages for missing work.

The study population had 3 specific subgroups: patients who were enrolled with back pain only (implant location T7) and back pain with unilateral pain or bilateral leg pain (implant location T7/T8/T9). All the enrolled population reported at least a 50% reduction in average pain relief. While all patients responded with significant improvements in pain, the best responses were seen in those who displayed some radicular component to their back pain. Historically, it has been shown that low back pain has been more difficult to treat than leg pain with SCS^22^, ^23^; however, this has been overcome with innovations in SCS, better understanding of stimulation targets, and improvements in patient selection.^23^, ^24^, ^25^ Even though patients with unilateral or bilateral leg pain may respond better to SCS, it is important to note that all patients in this study had a primary complaint of back pain, and if they had unilateral or bilateral leg pain, it was less severe than their back pain. While additional studies are needed to further clarify which patients are the best candidates for SCS implants, the results presented here demonstrate the safety and efficacy of BurstDR stimulation in NSLBP patients with paddle leads.

Paddle lead placements report low migration and revision rates.^21^, ^26^ Although rare, paddle leads may migrate, and there has been some concern about a higher risk of associated complications during replacement procedures.^21^ In the DISTINCT study, one patient underwent paddle revision, and two underwent paddle removal without additional complications. The DISTINCT paddle cohort reported a low adverse event rate, with 9/50 patients reporting an event. The study reports a 4% explant and 2% revision rate, which is considered low and acceptable as compared to the literature.^26^

The size and design of the paddle enable optimum coverage of the dorsal columns, resulting in precise stimulation and improved pain relief. Paddle electrodes are insulated dorsally, and the electrical current passes directly down to the spinal cord, making them energy efficient. Paddle leads have also been recommended as a rescue SCS implant after a failed or migrated percutaneous electrode implant.^26^ This flexibility in targeting specific areas of the spinal cord, theoretically allowing for better coverage of pain regions, may have been more pronounced with older tonic percutaneous leads.^27^ This study affords a unique opportunity to compare a matched patient group treated with percutaneous (Burst DR stimulation) and paddle implants contemporaneously.

A comparison of this cohort to the percutaneous lead group shows no significant difference in clinical performance for pain reduction, disability, and catastrophizing thoughts (*p* > 0.05). Both groups reported similar average pain scores (paddle‐2.2 vs. percutaneous‐2.5), ODI (paddle‐22.1 vs. percutaneous‐24.1), and PCS scores (paddle‐7.8 vs. percutaneous‐ 7.7). Likewise, the safety rates for each lead type are within range of reported lead fracture (1%) and migration rates (7%).^28^, ^29^

The increasing cost of inpatient healthcare has highlighted the cost savings afforded by ambulatory surgical centers (ASC).^28^, ^30^ These safety data affirm previously published reports that confirm comparable complication rates for both centers.^30^ In general, SCS is cost‐effective in the long‐term compared with continued medical management or reoperation.^30^, ^31^, ^32^

These data affirm that safety and performance do not significantly differ if a patient is trialed and implanted by a pain specialist or trialed by a pain specialist and implanted by a surgeon. Safety and performance rates for each lead type did not differ. Ultimately, the decision to use paddle leads for SCS should be made on a case‐by‐case basis, considering patient selection and risk–benefit profile to ensure optimal outcomes.

In conclusion, paddle leads represent a valuable treatment option in spinal cord stimulation for chronic pain management. The DISTINCT study shows that the use of paddle leads resulted in reduced pain and improved disability. These data are in line with outcomes presented in the literature and comparable with the DISTINCT percutaneous leads' outcomes. Careful patient selection and proper surgical technique are essential for optimizing efficacy and minimizing complications. In addition, the rate of adverse events is low, supporting a favorable risk–benefit ratio both in the hospital and ASC setting.

## CONCLUSION

The use of surgical paddle SCS leads in the treatment of chronic low back pain without corrective surgical options is safe and effective. The DISTINCT study patients' data demonstrate that the outcomes with paddle leads are comparable to those seen with percutaneous leads. In addition, this study provides a validation of the collaborative paradigm between interventional pain medicine and spine surgery specialists.

## AUTHOR CONTRIBUTIONS

UO analyzed the data and drafted the manuscript. All listed authors contributed to the data acquisition, data analysis, and draft editing. All authors have agreed to the final submitted version.

## FUNDING INFORMATION

This study was funded by Abbott Labs.

## CONFLICT OF INTEREST STATEMENT

Steven Falowski has consulting agreements with Medtronic, Abbott, Saluda, Vertos, Biotronik, CornerLoc, and Mainstay Medical. He has received research support from Aurora, Abbott, Mainstay, Medtronic, Vertiflex, CornerLoc, Saluda, and Biotronik. He also has stocks or stock options with BackStop Neural, SurgenTec, SynerFuse, Aurora Spine, Thermaquil, SPR Therapeutics, Saluda, CornerLoc, PainTeq, Stimgenics, Anesthetic Gas Reclamation, Neural Integrative Solutions, SpineThera, and Celeri. Robert M. Levy is an unpaid consultant for Abbott, Nalu, Biotronik, and Saluda Medical. Robert M. Levy has stock options with Nalu and Saluda. Udoka Okaro is an employee of Abbott. Julie Pilitsis receives grant support from Medtronic, Boston Scientific, Abbott, Focused Ultrasound Foundation, NIH‐NeuroBlueprint MedTech 5U54EB033650, and NIH R18EB036591. She is the medical advisor for Aim Medical Robotics and has stock equity. Timothy Deer has consulting agreements with Abbott, Saluda, SPR Therapeutics, Biotronik, and Nervonik. Timothy Deer has stocks or stock options with Saluda and Nervonik. He has received research support from Abbott, Saluda, and SPR Therapeutics. Timothy Deer is an Editorial Board member of pain practice journal and a co‐author of this article. To minimize bias, he was excluded from all editorial decision‐making related to the acceptance of this article for publication. Mehul Desai reports consulting fees from Abbott, SPR Therapeutics, and Nalu Medical and stock or stock options from SPR Therapeutics, Synerfuse, Virdio, and VYRSA. Chris Gilligan reports consulting fees from Mainstay Medical, Persica, Saluda, and Iliad Lifesciences and stock or stock options from Mainstay Medical. Chris Gilligan is an Editor‐in‐chief of Pain Practice and a co‐author of this article. To minimize bias, he was excluded from all editorial decision‐making related to the acceptance of this article for publication. Robert Heros reports consulting fees from Abbott, Mainstay Medical, Saluda Medical, Biotronik, and Boston Scientific and support for attending meetings from Mainstay Medical and participated on a data safety monitoring board/advisory board for Biotronik. Jessica Jameson reports consulting fees from Boston Scientific, Nevro, Saluda, Abbott, and SI Bone and payment or honoraria from Nevro, Boston Scientific, Saluda, Abbott, and Medtronic. Chi Lim reports consulting fees from Synthes, Medtronic, Abbott, Implanet. Michael J. Dorsi has consulting agreements with Abbott, Nevro, Globus, Camber, LifeSpine, Kyocera, and Met One. James J. Yue reports consulting income and grant support from Abbott. All other authors report no conflict of interest.

## PATIENT CONSENT

Consent was obtained from all potential patients prior to enrollment. The study is conducted in accordance with the US Code of Federal Regulations and the World Medical Association Declaration of Helsinki.

## CLINICAL TRIAL REGISTRATION

The study is registered on ClinicalTrials.gov (NCT04479787).

## Supporting information


Data S1.

## Data Availability

Data are included within the manuscript.
