# Supplementary material for: Paddle leads for the treatment of nonsurgical back pain—The DISTINCT study
Source: Pain Pract. 2025 Apr 23;25(5):e70033. doi: 10.1111/papr.70033 (PMC12015649; doi:10.1111/papr.70033)
Supplement: Supplementary file 1 — Data S1. [file PAPR-25-0-s001.docx]

**Supplemental Data**

**Table S1: Outcome tables stratified by pain location**

| **Pain location** | **N** | **Implant location** | **Pain score** | | | **ODI** | | |
| --- | --- | --- | --- | --- | --- | --- | --- | --- |
|  |  |  | **Baseline** | **6M** | **12M** | **Baseline** | **6M** | **12M** |
| Back pain Only | 16 | T7 | 7.6 ± 1.3 (16)  [7.0, 8.3] | 2.4 ± 1.7 (14)  [1.5, 3.4] | 3.5 ± 2.8  (12)  [1.7, 5.3] | 51.3 ± 14.8  (16)  [43.4, 59.1] | 20.3 ± 11.6  (13)  [13.3, 27.3] | 25.4 ± 16.4  (12)  [15.0, 35.9] |
| Back pain and unilateral leg pain | 16 | T7, T8, T9 | 7.9 ± 1.0  (16)  [7.4, 8.5] | 1.9 ± 1.7  (15)  [0.9, 2.8] | 1.5 ± 1.5  (15)  [0.7, 2.4] | 54.4 ± 14.2  (16)  [46.9, 61.9] | 19.3 ± 12.6  (14)  [12.0, 26.6] | 20.9 ± 8.9  (13)  [15.5, 26.3] |
| Back pain and bilateral leg pain | 18 | T7, T8, T9 | 8.1 ± 1.3  (18)  [7.4, 8.7] | 1.9 ± 1.5  (18)  [1.2, 2.6] | 1.8 ± 1.8  (17)  [0.9, 2.8] | 57.3 ± 16.2  (18)  [49.2, 65.4] | 20.1 ± 17.2  (18)  [11.6, 28.6] | 20.7 ± 14.7  (17)  [13.2, 28.3] |

Summaries are Mean ± SD (N) [95% CI]

**Table S2: Outcome tables for the DISTINCT population stratified by lead type**

|  | **Paddle** | | | **Percutaneous Leads** | | | **P** |
| --- | --- | --- | --- | --- | --- | --- | --- |
|  | **Average** | **STD** | **N** | **Average** | **STD** | **N** |  |
| **NRS** | 2.2 | 2.2 | 44 | 2.7 | 2.1 | 54 | 0.237 |
| **ODI** | 22.1 | 13.6 | 42 | 25.8 | 13.6 | 50 | 0.205 |
| **PCS** | 7.8 | 9.9 | 44 | 7.5 | 10.6 | 53 | 0.890 |
